# A randomised clinical trial to evaluate the effects of *Plantago ovata* husk in Parkinson patients: changes in levodopa pharmacokinetics and biochemical parameters

**DOI:** 10.1186/1472-6882-14-296

**Published:** 2014-08-12

**Authors:** M Nelida Fernandez-Martinez, Luis Hernandez-Echevarria, Matilde Sierra-Vega, M Jose Diez-Liebana, Angela Calle-Pardo, Demetrio Carriedo-Ule, Ana M Sahagún-Prieto, Anna Anguera-Vila, Juan Jose Garcia-Vieitez

**Affiliations:** Area de Farmacologia, Instituto de Biomedicina (IBIOMED), Universidad de Leon, 24071 Leon, Spain; Complejo Hospitalario Universitario de Leon, 24071 Leon, Spain; Laboratorios Rottapharm, 08033 Barcelona, Spain

**Keywords:** Levodopa, Parkinson patients, Pharmacokinetics, Biochemical parameters, Plantago ovata husk

## Abstract

**Background:**

Plantago ovata husk therapy could be used in patients with Parkinson disease to reduce the symptoms of gastrointestinal disorders, but it is important to know whether this compound modifies levodopa pharmacokinetics. The maintenance of constant plasma concentrations of levodopa abolishes the clinical fluctuations in parkinsonian patients. The aim of this randomised clinical trial was to establish the influence of the fiber Plantago ovata husk in the pharmacokinetics of levodopa when administered to Parkinson patients well controlled by their oral medication.

**Methods:**

To evaluate the effects of this fiber on several biochemical parameters. 18 volunteers participated in the study and received alternatively two treatments (Plantago ovata husk or placebo) with their usual levodopa/carbidopa oral dose. On days 0 (initial situation), 14 and 35 of the study, blood samples were taken to assess levodopa pharmacokinetics and to determine biochemical parameters.

**Results:**

Levodopa C_max_ was very similar in the initial situation (603.2 ng/ml) and after placebo administration (612.0 ng/ml), being slightly lower (547.8 ng/ml) when Plantago ovata husk was given. AUC was very similar in the three groups: initial situation.- 62.87 μg.min/ml, fiber treatment.- 64.47 μg.min/ml and placebo treatment.- 65.10 μg.min/ml. Fiber reduced significantly the number of peaks observed in the levodopa concentrations, maintaining concentrations more stable. No significant differences were found in total cholesterol, LDL-cholesterol and triglycerides with the administration of Plantago ovata husk.

**Conclusions:**

Plantago ovata husk administration caused a *smoothing* and *homogenization* of levodopa absorption, providing more stable concentrations and final higher levels, resulting in a great benefit for patients.

**Trial registration:**

EudraCT2006-000491-33

## Background

Parkinson’s disease remains one of the most common neurodegenerative diseases, affecting 1% of the population over 65 [1]. Autonomic disorders are often seen in idiopathic Parkinson, especially in the advanced stage of the disease. Disturbances of the gastrointestinal tract are considered to be the most frequent autonomic disorders, including abnormal salivation, difficulty of swallowing (dysphagia), early feeling of satiety (abdominal bloating-distension), disorders of gastric emptying and constipation [2–4]. These symptoms may affect the quality of life of Parkinson’s patients.

Constipation has been reported in over 50% of patients with Parkinson’s disease attending a movement disorders clinic [2]. In the elderly, constipation may correlate with decreased physical activity, a low fluid intake, a diet poor in fiber, the sedentary life style and also to the illness itself [5]. Prokinectic drugs may help reduce the symptoms of gastrointestinal dismotility, but the side effects preclude their prolonged use [6].

Plantago ovata husk (ispaghula husk) is a viscous water-soluble fiber that has been used as a bulk laxative with a good safety record. This fiber could be a good alternative to help to solve constipation problems in patients with Parkinson’s disease. However, it is important to know whether this compound modifies levodopa pharmacokinetics. Fiber can retain a part of the dose administered; it also can modify gastric emptying as well as the absorption conditions in the small bowel and the presystemic clearance of the drug.

The influence of Plantago ovata husk on levodopa pharmacokinetics could be clinically relevant due to the special kinetic features of this antiparkinsonian drug.

Levodopa has a peculiar pharmacokinetics, characterised by an extensive presystemic metabolism, a rapid absorption in the proximal small intestine and a very short plasma half-life due to its rapid metabolism [7].

Maintenance of constant plasma concentrations of levodopa largely abolishes the clinical fluctuations in parkinsonian patients [7–10], indicating that delivery of levodopa to the striatum is a critical determinant of clinical response [11].

Dietary factors can modify the rate and extent of levodopa absorption. It is well established, for example, that its absorption can be reduced or retarded by concomitant food intake [12, 13]. On the other hand, aromatic and branched chain amino acids of concomitant protein rich meals may compete with levodopa for the carrier system across the intestinal mucosa, directly affecting levodopa absorption [14].

Therefore, an understanding of the pharmacokinetics of any drug is crucial for the establishment of its optimal therapeutic regimen. This assumes a special importance with levodopa [14].

The aim of the present study was to establish the influence of the fiber Plantago ovata husk (that provides the aqueous solution a high viscosity) in the pharmacokinetics of levodopa when administered to Parkinson patients well controlled by their oral medication. We also evaluated the effects of this fiber on several biochemical parameters such as glucose, cholesterol, uric acid, etc.

## Methods

### Patients and treatment

The study carried out was a prospective, aleatory crossed and double-blind randomised clinical trial (EudraCT2006-000491-33; clinicaltrials.gov/ct2/show/NCT00507715). We studied 18 patients (10 men, 8 women) with idiopatic Parkinson disease attending the Neurology Service of the Hospital Universitario de Leon, Spain, whose sympthoms were controlled by levodopa/carbidopa oral medication. The recruitment of participants took place between April and May 2006, and the trial developed between September and November 2006.

Inclusion criteria were: (a) patients with idiopathic Parkinson disease whose sympthoms were controlled by levodopa/carbidopa oral medication; (b) at least 3 months of levodopa treatment; (c) between 60 and 80 years of age. Exclusion criteria were: (a) patients participating in other clinical trial or that have participated in the last month; (b) allergy or contraindication to *Plantago ovata* husk; (c) chronic renal failure or hepatic disorders; (d) psychiatric disorders; (e) patients with diabetes mellitus or in treatment with oral hypoglcemic agents.

The study was approved by the Ethical Committee of the Hospital de León as well as the Spanish Medicines Agency (AEMPS). All participants provided written informed consent prior to enrolment into the trial. At the time of examination, the mean age was 69.8 ± 4.2 years, the mean disease duration was 1.3 ± 0.5 years, and the mean duration of levodopa treatment was 0.7 ± 0.5 years.

**Figure 1:**
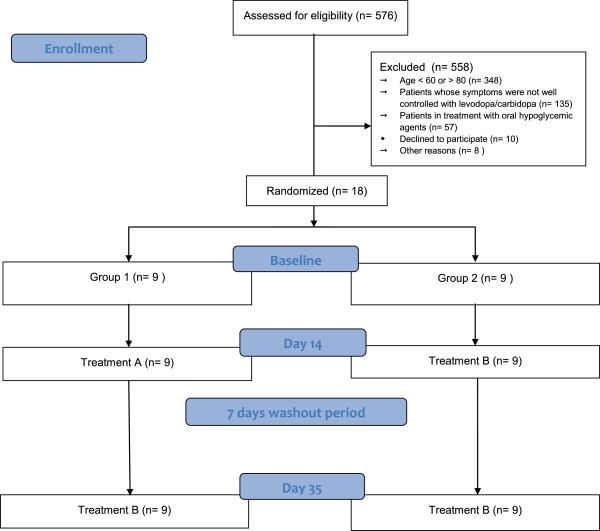
**Flow chart of the study participants.**

Volunteers were randomly divided into two groups of 9 patients each (Table 1). Both groups received alternatively two treatments according to the diagram included in Figure 1: treatment A, administration of Plantago ovata husk; and treatment B, administration of placebo. To generate the random allocation, a numbered list of the participants was created and an Excell aleatory number generator was used.

**Table 1:** **Baseline demographic and clinical characteristics of the participants**

	Group 1 (n = 9)	Group 2 (n = 9)
**Sex (Men/Women)**	(5/4)	(5/4)
**Age (Mean ± SD) (years)**	68.7 ± 3.1	70.3 ± 4.3
**Disease duration (Mean ± SD) (years)**	1.4 ± 0.6	1.3 ± 0.4
**Duration of levodopa treatment (Mean ± SD) (years)**	0.7 ± 0.3	0.8 ± 0.5

During treatment A (Plantago ovata husk administration), volunteers received their usual levodopa/carbidopa oral dose (100/25 mg), (Sinemet®, MSD) three times a day and, immediately before, 3.5 g *Plantago ovata* husk (Plantaben®, Rottapharm S.L., Barcelona, Spain) dispersed into 200 ml water. *Plantago ovata* husk was manufactured as a palatable, orange-flavoured, sugar-free product, distributed in sachets of 5 g each, containing 3.5g of Plantago ovata husk. The other 9 patients (treatment B) received placebo instead of fiber. Patients followed these treatments for 14 days, and after a wash-out period of 7 days, the other treatment (A or B) was given.

To assess levodopa pharmacokinetics, on days 0, 14 and 35 of the study, volunteers came up to the Neurology Service of the Hospital Universitario de Leon at 8:00 AM following an overnight fast. After taking their medication, blood samples were obtained through an intravenous catheter at 0, 10, 20, 30, 45, 60, 90, 120, 180, 240 and 300 min after drug administration. At time 0 an additional sample was collected to determine biochemical parameters: glucose, triglycerides, total cholesterol, LDL-cholesterol, HDL-cholesterol, uric acid, creatinin.

Immediately after blood sample collection, plasma was separated by centrifugation and stored at –20°C until analysis.

The results obtained on day 0 (initial situation) served to asses patients conditions before starting the treatment with Plantago ovata husk or placebo. At the end of the visit, the Plantago ovata husk or placebo treatment necessary for the first 14 days of the study was given to the volunteers.

On day 14, the patients brought the Plantago ovata husk or placebo packing (full or empty) given on day 0 to evaluate therapeutic compliance, and received the Plantago ovata husk or placebo treatment necessary for the next 14 days of the study (from day 21 to 35, after a 7 days wash-out period). On the final visit (day 35), the full or empty packing were also collected.

### Levodopa determination

Levodopa extraction from plasma samples was carried out by using a catecholamine kit (Chromsystems®) and was quantified by HPLC with electrochemical detection.

The mobile phase consisted of 50 mM sodium dihydrogenphosphate buffer adjusted to pH 2.9 with 1 M orthophosphoric acid containing 250 mg/l heptanesulphonic acid and 80 mg/l EDTA and methanol (90:10, v/v). This mobile phase was pumped at a flow rate of 1 ml/min.

The analytical column was a 25 cm × 4.6 mm I.D. stainless-steel column, packed with Spherisorb ODS-2 (5 pm particle size, Waters Chromatography SA, Madrid, Spain) and the potential applied was 500 mV. Interday and intraday accuracy and precision were within 10%.

### Pharmacokinetic studies

Pharmacokinetic analysis was performed based on a non-compartmental description of the data observed.

Maximum plasma levodopa concentration (C_max_) and the time to reach maximum concentration (t_max_) were read directly from the individual plasma concentration-time curves.

The WinNonlin® computer program and formulae described by Gibaldi and Perrier [15] were used to calculate the model-independent pharmacokinetic parameters: the elimination rate constant (λ), the area under the plasma concentration-time curve (AUC), the clearance (Cl/F), the volume of distribution at steady state (V_ss_/F), the half-life associated with λ phase (t_1/2λ_), the area under the first moment curve (AUMC), and the mean residence time (MRT).

Levodopa concentration was also determined in the sample taken at time 0 and it was considered as minimum plasma levodopa concentration (C_min_). In addition, a qualitative pharmacokinetic variable was studied: the number of peaks observed in the levodopa concentration-time curve.

### Statistical evaluation

The following formula was used to estimate the sample size:


Calculations were performed using 80% power, a 5% significance level and a standard deviation/precision ratio of 0,75.

All pharmacokinetic and biochemical parameters were calculated for each volunteer and presented as arithmetic mean ± standard deviation (mean ± SD). Data were analysed using the Skewness test (to determine the normality) and Cochran test (to determine the uniformity of the variance).

The statistical analysis taking into account subject as a random effect and the period effect was done. When the data were normal and there was uniformity in the variance, analysis of variance (ANOVA with repeated measures) was carried out and the paired *t* test was used to determine differences between data sets. When the data were not normal and/or there was not uniformity in the variance, Friedman test was employed and the Wilcoxon test was used to determine differences between data sets.

To evaluate differences in the number of peaks observed in the concentration versus time curves (one peak or more than one peak), Pearson’s Chi-squared was employed, or Fisher test when the first could not be used. P ≤ 0.05 was used as the level of significance for all analyses.

## Results

**Figure 2:**
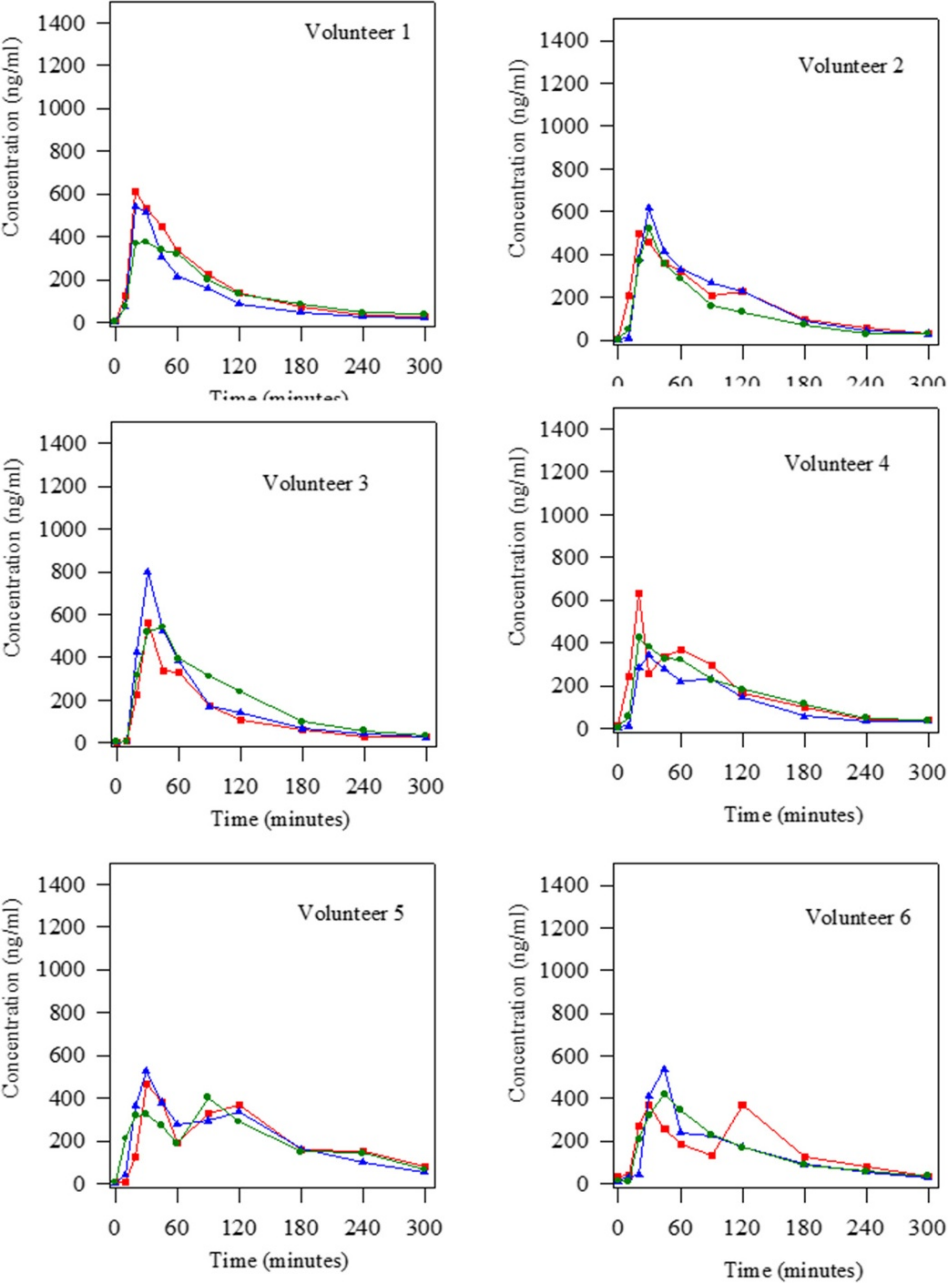
**Levodopa concentrations obtained alter the oral administration of 100:25 mg/kg levodopa/carbidopa in the three groups studied (initial situation blue triangle; treatment with Plantago ovata husk green circle and treatment with placebo red square) to 18 Parkinson patients.** Volunteers 1 to 6.

**Figure 3:**
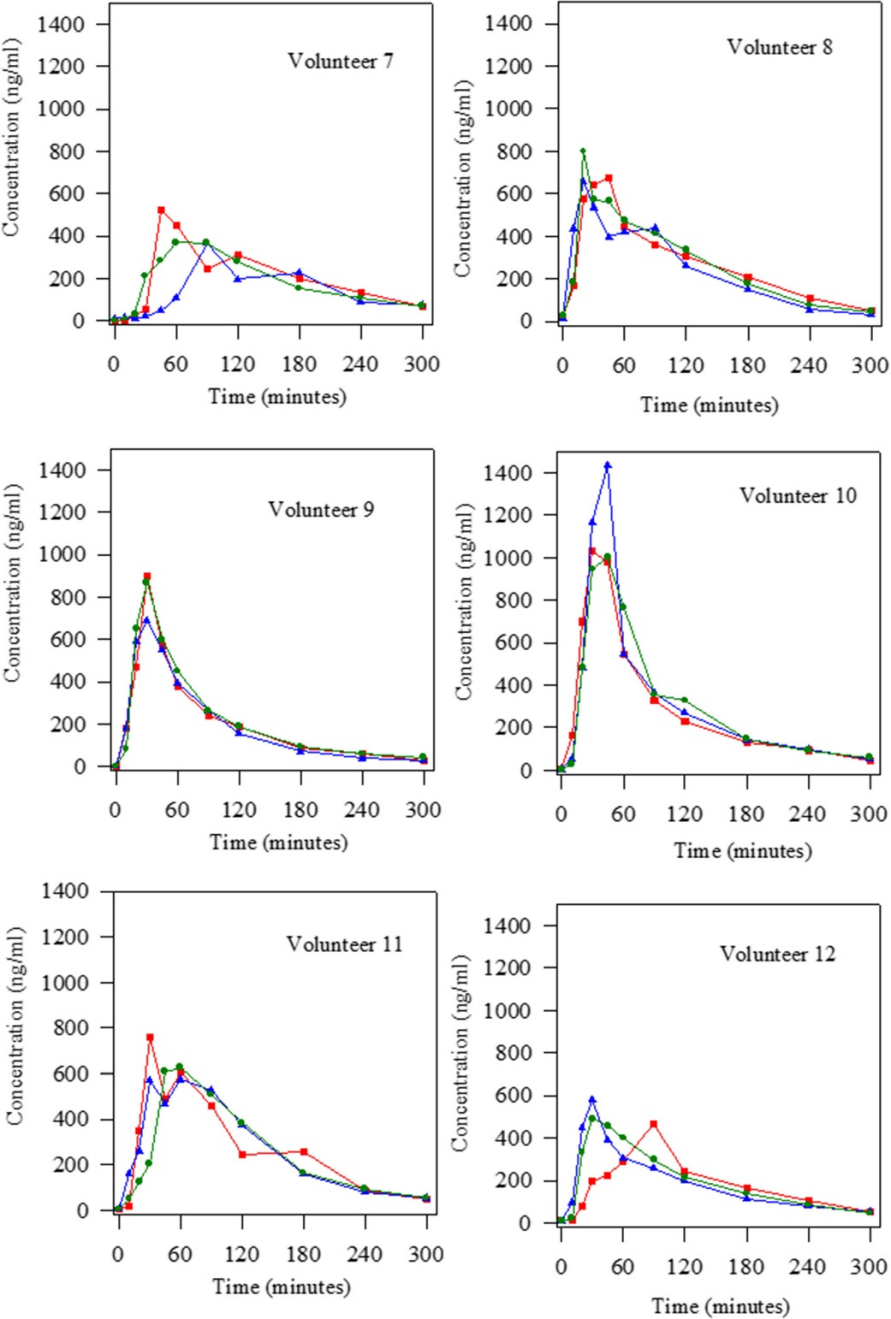
**Levodopa concentrations obtained alter the oral administration of 100:25 mg/kg levodopa/carbidopa in the three groups studied (initial situation blue triangle; treatment with Plantago ovata husk green circle and treatment with placebo red square) to 18 Parkinson patients.** Volunteers 7 to 12.

**Figure 4:**
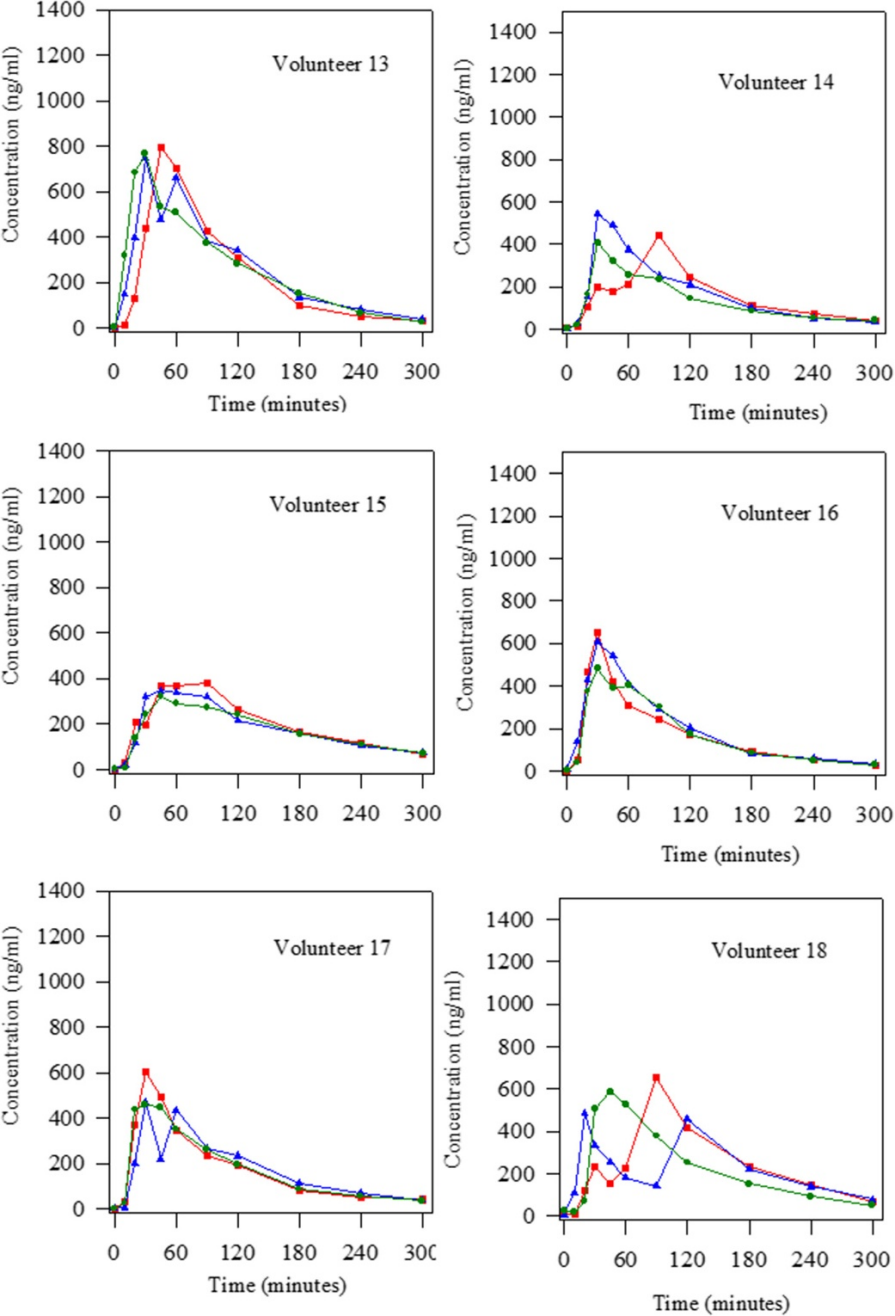
**Levodopa concentrations obtained alter the oral administration of 100:25 mg/kg levodopa/carbidopa in the three groups studied (initial situation blue triangle; treatment with Plantago ovata husk green circle and treatment with placebo red square) to 18 Parkinson patients.** Volunteers 13 to 18.

All the patients enrolled in the study completed the two phases of the clinical trial. The median obtained for therapeutic compliance was 100%, with a minimum of 98% and a maximum of 100%.The plots of plasma levodopa concentration as a function of time obtained in each patient for the three treatments studied (initial situation, Plantago ovata husk treatment and placebo treatment) are shown in Figures 2, 3 and 4.

The non-compartmental pharmacokinetic parameters obtained after the administration of 100:25 mg/kg levodopa/carbidopa and in the presence of *Plantago ovata* husk or placebo are summarized in Table 2.

**Table 2:** **Pharmacokinetic parameters obtained alter the oral administration of 100:25 mg/kg levodopa/carbidopa in the three groups studied (initial situation; treatment with Plantago ovata husk and treatment with placebo) to 18 Parkinson patients**

	Initial situation		Treatment A (Plantago ovata husk)		Treatment B (Placebo)	
Parameters	Mean ± SD	CV(%)	Mean ± SD	CV(%)	Mean ± SD	CV(%)
**t**_**max**_ **(min)**	35.83 ± 16.91	47.20	39.72 ± 17.19	43.28	36.17 ± 26.30	59.56
**C**_**max**_ **(ng/ml)**	603.2 ± 242.4	40.19	547.8 ± 192.6	35.16	612.0 ± 176.6	28.86
**AUC (μg.min/ml)**	62.87 ± 15.77	25.09	64.47 ± 15.27	23.69	65.10 ± 14.33	22.01
**λ (min** ^**-1**^ **)**	0.0096 ± 0.0018	18.87	0.0088 ± 0.0020	22.54	0.0097 ± 0.0018	18.40
**V**_**ss**_ **/F (l)**	0.1845 ± 0.0628	34.05	0.1929 ± 0.0521	27.04	0.1699 ± 0.0468	27.58
**Cl/F (l/min)**	0.0017 ± 0.0004	25.98	0.0016 ± 0.0004	23.89	0.0016 ± 0.0004	22.52
**AUMC (μg.min** ^**2**^ **/ml)**	7881.7 ± 2630.3	33.37	8313.7 ± 2284.4	27.48	8327.1 ± 2651.9	31.85
**MRT (min)**	125.1 ± 29.9	23.93	129.2 ± 21.7	16.78	126.6 ± 24.2	19.13
**C**_**min**_ **(ng/ml)**	6.02 ± 3.41	56.70	6.31 ± 7.10	112.58	7.34 ± 7.98	108.71
**t**_**1/2λ**_ **(min)**	75.2 ± 16.0	21.27	81.9 ± 15.3	18.70	74.0 ± 16.9	22.86

The values obtained for C_max_ were very similar in the initial situation (603.2 ± 242.4 ng/ml) and after the administration of placebo (612.0 ± 176.6 ng/ml). This parameter was slightly lower (547.8 ± 192.6 ng/ml) when Plantago ovata husk was given, although there were no significant differences between groups (ANOVA).

Regarding AUC, the mean value determined for this parameter was very similar in the three groups: 62.87 ± 15.77 μg.min/ml in the initial situation, 64.47 ± 15.27 μg.min/ml after fiber treatment and 65.10 ± 14.33 μg.min/ml after placebo treatment.

Levodopa absorption was slightly slower in the presence of Plantago ovata husk (t_max_ = 39.72 min) than in the initial situation (t_max_ = 35.83 min) or after placebo administration (t_max_ = 36.17 min). However, no significant differences were found between these values. In addition, there were no significant differences in any of the pharmacokinetic parameters studied.

Regarding the number of peaks observed in the levodopa concentration-time curves, a very important fact was found. It can be seen (Figures 2, 3 and 4) that there were, respectively, 8, 2 and 9 patients that showed more than one peak in the initial situation, after fiber treatment and when placebo was administered. Statistical analysis revealed that there were significant differences between treatment with fiber and the other two groups studied (initial situation and placebo). So, fiber clearly reduced the number of peaks maintaining levodopa concentrations more stable.

As we have mentioned, at time 0, an extra sample was taken to determine biochemical parameters. Table 3 includes the mean value for these parameters on day 0 (initial situation) and after fiber or placebo treatment.

**Table 3:** **Pharmacokinetic parameters obtained alter the oral administration of 100:25 mg/kg levodopa/carbidopa in the three groups studied (initial situation; treatment with Plantago ovata husk and treatment with placebo) to 18 Parkinson patients**

	Initial situation	Treatment A (Plantago ovata husk)	Treatment B (Placebo)
**Glucose (mg/100 ml)**	105.50 ± 10.65	96.56 ± 10.62	98.06 ± 13.40
**Urea (mg/100 ml)**	42.96 ± 9.50	43.43 ± 11.76	45.93 ± 12.50
**Creatinine (mg/100 ml)**	0.89 ± 0.17	0.85 ± 0.15	0.86 ± 0.15
**Tryglicerides (mg/100 ml)**	94.83 ± 42.39	84.33 ± 29.28	94.11 ± 43.73
**Total cholesterol (mg/100 ml)**	214.39 ± 36.92	191.89 ± 27.73	209.50 ± 36.65
**AST (GOT) (UI/l)**	21.22 ± 4.49	19.94 ± 4.47	20.33 ± 4.34
**ALT (GPT) (UI/l)**	25.67 ± 8.98	25.06 ± 9.51	26.00 ± 9.29
**Alkaline phosphatase (UI/l)**	131.44 ± 43.91	124.00 ± 40.68	126.00 ± 44.13
**GGT (UI/l)**	17.06 ± 6.55	16.00 ± 6.15	19.85 ± 10.63
**LDL (mg/100 ml)**	135.18 ± 29.30	117.67 ± 22.23	132.32 ± 33.00
**HDL (mg/100 ml)**	60.17 ± 13.90	57.33 ± 15.91	58.33 ± 12.34

When fiber was administered, there was a slight reduction in total cholesterol (191.9 mg/100 ml) in comparison with the initial situation (214.4 mg/100 ml) and placebo treatment (209.5 mg/100 ml). The mean values for tryglicerides and LDL-cholesterol were also lower (10 and 15%, respectively). However, no significant differences were found between the three groups studied in any of the biochemical parameters evaluated.

## Discussion and conclusions

Levodopa remains to be one of the main drugs in the treatment of Parkinson’s disease. Studies on the concentration-effect relationship of levodopa in Parkinson’s disease have established that it is beneficial to maintain stable plasma concentrations of levodopa [7–10].

Several authors have studied the pharmacokinetics of levodopa in patients with Parkinson’s disease. After the administration of 100 mg levodopa combined with a dopa decarboxylase inhibitor, the values obtained for C_max_ were next to 1000 ng/ml [16–18]. In our study, in the initial situation of patients, the data ranged from 343.1 to 1433.9 ng/ml, so, in some patients the values were similar to that found by other authors and in others it was lower. A similar result was obtained for t_max_. This parameter ranged from 20 to 90 minutes as in other studies [10, 17, 19]. However, the mean AUC value (62.87 μg.min/ml) was slightly lower than that found by other authors using the same dose of levodopa: 98.9 μg.min/ml [16], 99.7 μg.min/ml [18], 117.0 μg.min/ml [17].

In the present study, we evaluated the influence of Plantago ovata husk in the pharmacokinetics of levodopa in well controlled Parkinson patients. The results obtained showed that after Plantago ovata husk administration no significant differences were obtained for the pharmacokinetic parameters evaluated. However, its absorption increased slightly, with more stable concentrations and final higher levels. The lack of significant differences can be explained by the great differences observed in the pharmacokinetic behaviour of levodopa in each patient. Due to this fact, it is very important to evaluate each patient in separate, and, as the figures show, in most of them, the administration of fiber improved the concentration-time curve for levodopa as well as its pharmacokinetics: the levels are more stable, with lower maximum concentrations and higher final levels.

Considering the qualitative pharmacokinetic variable studied: presence of one or more than one peak in the concentration-time curve, it was clearly demonstrated that Plantago ovata husk treatment reduced the number of peaks. Other authors [20–22] also observed the presence of more than one peak in levodopa concentration-time curves, and its frequency (40-45%) was similar to that observed in this study. After Plantago ovata husk administration, only 11% patients showed more than one peak in the curve, while in the initial situation the percentage was 44% and with placebo 50%. So, it was proved that this fiber, Plantago ovata husk, helps to maintain more stable levodopa concentrations, a very important fact in Parkinson patients.

On the other hand, no significant differences were found in total cholesterol, LDL-cholesterol and triglycerides although the levels were slightly lower in the presence of Plantago ovata husk.

Other authors have demonstrated the beneficial effects of fiber lowering cholesterol, triglycerides and glucose [23–25]. Probably, we have not found a reduction in biochemical parameters due to the short time of treatment with fiber (14 days).

When the data obtained for the three situations studied (initial situation, fiber administration and placebo administration) are analyzed simultaneously, we can see important inter- and intra-individual differences regarding levodopa pharmacokinetics. For several volunteers, the levodopa concentration-time curves are different on day 0 (initial situation) and after placebo treatment, showing important intra-individual differences. Although levodopa kinetics was altered in the volunteers, the study gives important information due to all patients received both treatments, and so, pharmacokinetics was followed in every patient. Knowledge of individual patients’ levodopa behavior can contribute to rationalize drug treatment according to disease progression [10].

In recent years, researchers’ attention has mainly focused on overcoming levodopa treatment complications by optimizing drug pharmacokinetics [9, 26].

The knowledge of the main determinants of levodopa clinical pharmacokinetics is important in rationalising drug prescription at the start of the treatment but becomes essential with the advance of Parkinson’s disease [27]. As the disease progresses, the synaptic dopamine concentrations and clinical effect become strictly dependent on plasma levodopa concentrations, and the factors affecting peripheral levodopa pharmacokinetics become critical to the therapeutic response [9, 28].

The levodopa plasma half-life is very short, resulting in marked plasma drug concentration fluctuations which are matched, as the disease progresses, with swings in the therapeutic response (“wearing-off” phenomena). “Wearing-off” phenomena can be also associated to the more advanced disease stages with a “negative”, both parkinsonism-exacerbating and dyskinetic effect of levodopa at subtherapeutic plasma concentrations. Dyskinesias may be also related to high-levodopa, excessive plasma concentrations [9].

On the other hand, absorption of levodopa in the proximal small intestine depends on gastric emptying, which is erratic and may be slowed in Parkinson’s disease.

In a study carried out by Djaldetti *et al*. in 1995 [29], these authors concluded that “delayed-on” (prolonged latencies to onset) phenomenon and “non-on” (treatment failure) phenomenon are related to alterations of the gastrointestinal transit time and absorption of levodopa.

Previous studies carried out in experimental animals [30–33] showed that the administration of Plantago ovata husk with levodopa/carbidopa treatment improved levodopa pharmacokinetics. Fiber administration reduced the maximum plasma concentrations (lower adverse effects) and provided higher final concentrations (longer effect).

In the present study we have clearly demonstrated that the fiber Plantago ovata husk contributed to obtain more stable levodopa concentrations, with a reduction in the number of patients with more than one peak in the concentration-time curve. Plantago ovata husk administration caused a *smoothing* and *homogenization* of levodopa absorption, providing more stable concentrations and final higher levels, resulting in a great benefit for patients.

Taking into account the results obtained, and although a long-term study may be carried out, it will be beneficial for Parkinson patients to take Plantago ovata husk with their usual levodopa treatment since the beginning of the illness, in order to improve levodopa pharmacokinetics and the response to this drug.
